# Development of Genomic Sciences in Mexico: A Good Start and a Long Way to Go

**DOI:** 10.1371/journal.pcbi.0030143

**Published:** 2007-09-28

**Authors:** Rafael Palacios, Julio Collado-Vides

**Affiliations:** University of California San Diego, United States of America; University of California Berkeley, United States of America

## Introduction

The most important revolutionary frontier of the biological sciences in the present century is derived from the knowledge of the complete genomic information of different organisms. The discoveries derived from genomics are generating a new paradigm in biology, substituting gene-centered biology with a new level of integration: genome-centered biology. This new concept is the basis for potential developments of great social impact in different fields such as medicine, agriculture, and industry, to mention only a few.

The knowledge necessary for this new area is derived from different disciplines, mainly mathematics, computer sciences, and biology. Here we refer to the integration of these disciplines as “genomic sciences.” Rather than intending a comprehensive review of the state of the art in Mexico, we will discuss key aspects that allowed the initiation of, and that will certainly shape the development of, genomic sciences in this country.

## Origins

A pioneering effort of genome sciences in Mexico was the participation of a Mexican research group in the Escherichia coli genome project [1]. This participation was through the Computational Genomics Program of the Nitrogen Fixation Research Center (CIFN) of the National Autonomous University of Mexico (UNAM), and consisted of the annotation of upstream regulatory elements and operons in the E. coli genome. This was the first bacterial genome, among still few, with predictions on operons and upstream regulatory elements.

The first large-scale sequencing project in Mexico was also performed at CIFN and consisted of the determination of the 370-kb nucleotide sequence of the symbiotic plasmid of Rhizobium etli [2]. R. etli participates in a nitrogen-fixing symbiosis with the common bean plant, Phaseolus vulgaris. The bean plant diversified from Mesoamerica, and *R. etli,* which was first reported by a Mexican team, is found all over Mexico and presents a high degree of genetic diversity. This achievement was the basis for the support by the National Council of Science and Technology (CONACYT; http://www.conacyt.mx/) of a project entitled “Development of Genomic Sciences in Mexico: The Genome of Rhizobium etli as a Model System.” The success of this project resulted in the first complete genome sequencing performed in Mexico [3] and represented a key step in the development of genomic sciences at UNAM and in the country as a whole.

## Research in Genomics

Currently, there are several genomic and postgenomic projects ongoing in different Mexican institutions. The genome sequencing of R. etli stimulated several projects at the Center for Genomic Sciences at UNAM (CCG; http://www.ccg.unam.mx/), formerly the CIFN (see below). Such projects are related to the evolution and microevolution, the transcriptomics and proteomics, the dynamics, and the comparison of syntenic genes as a commonly evolved group of the genomes of rhizobial organisms [4–6]. The study of the dynamics of the R. etli genome recently led to a research line aimed at better understanding the dynamics of the human genome [7]. Another project at CCG is related to the functional genomics of the host legume of *R. etli,* the common bean plant P. vulgaris. This project, known as “Phaseomics,” is being pursued by an international consortium initially proposed by CCG and the University of Geneva [8]. An ambitious grant proposal involving different institutions from Mexico, the United States, and Colombia has recently been submitted to CONACYT to determine the DNA sequence of the genome of P. vulgaris.

Another major genomic project of UNAM is related to the human parasite Taenia solium. At the first stage, the DNA sequences of a large number of expressed sequence tags will be obtained, and, subsequently, the project intends to produce a high-quality draft of the whole genome. This project is driven by a consortium of laboratories, and at the moment includes about 30 researchers [9].

CCG has for years worked on the computational representation and analysis of the regulatory network of *E. coli,* curated into RegulonDB and EcoCyc, and studied promoters, regulation of initiation of transcription, operons, and regulatory proteins [10]. The Institute of Biotechnology (IBT; http://www.ibt.unam.mx/) at UNAM is also very active in bioinformatics research, notably working in computational predictions of riboswitch-like elements and curvature regions in bacterial genomes, the development of gene context analysis, and the evolution of metabolism and protein structure and function [11–13].

Several genomic and postgenomic studies involve industrial and pathogenic E. coli [14]. Different Mexican institutions participate in such projects; these include IBT, the Institute of Ecology (http://www.ecologia.unam.mx/) at UNAM, and the School of Medicine at UNAM.

A group at the Institute of Ecology is participating in exciting metagenomic projects. The major interest of this Mexican team is focused in Cuatro Ciénegas, an oasis in the desert of the state of Coahuila, in north Mexico, which constitutes a very ancient ecological niche of extreme conditions, with communities from the Cambrian Period [15]. Another group performs theoretical modeling and experimental validation of the regulatory network governing flower development in *Arabidopsis* [16].

In the School of Sciences at UNAM, there is a group that has a long tradition of studying the origin of life, and that has been working on several aspects of microbial evolution such as lateral gene transfer and early origin of oxidative damage protection, among others [17].

There has been a tradition of research in biomathematics and theoretical biology spread throughout UNAM, by physicists, mathematicians, biologists, and theoreticians. Recently, this is reflected in the interest in establishing a new center devoted to complex systems, in Cuernavaca.

In addition to UNAM, another major research institution in Mexico, the Center for Research and Advanced Studies (CINVESTAV; http://www.cinvestav.mx/) of the National Polytechnic Institute, is making important contributions to genomic sciences. This institution recently launched the National Laboratory of Genomics for Biodiversity of Mexico (LANGEBIO; see below) with state-of-the-art facilities and a core of internationally known researchers. Its major project is the sequencing of all the genes of maize and the determination of their main functions, but its research also expands to many other plant models including *Arabidopsis,* bean, chile, and tomatillo [18].

The health sector in Mexico has also participated very actively in genomic research, such as in the study of *Trichoderma,* in particular in institutions belonging to the Mexican National Institutes of Health. Several projects relate to the discovery and study of the incidence of genes involved in common disorders in the Mexican population, such as type 2 diabetes, obesity, and atherosclerosis [19]. Of particular importance has been the launching of the National Institute of Genomic Medicine (INMEGEN; http://www.inmegen.gob.mx/; see below). This institute has started an ambitious project to map the genome of the Mexican mestizo population. Researchers hope that the “Mexican Hapmap” project will lead to the creation of medical applications that will benefit the country and eventually help to stratify the Mexican population for clinical trials and personalized medicine [20].

With regard to genomic resources, high-technology facilities for bioinformatics DNA sequencing, transcriptomics, and proteomics have been established at the major institutions participating in the development of genomic sciences in Mexico: UNAM, CINVESTAV, and the Mexican National Institutes of Health.

## New Institutions and Funding

New institutions that will be key elements for the future development of genomic sciences in Mexico are now being established. In 2004, CIFN was transformed into CCG, with the mission of integrating research and education in order to participate, together with other institutions, in the development of genomic sciences at UNAM and elsewhere in the country. The National Institute of Genomic Medicine was created in 2004 as one of the Mexican National Institutes of Health. Its general mission is to contribute to the health of the Mexican population through frontier scientific research and the development of scientists at the Ph.D. level in the field of genomic medicine. The CINVESTAV launched the National Laboratory of Genomics for Biodiversity of Mexico in 2005. The mandate of this institution is to do research and graduate training in genomics for the protection and sustainable use of national biodiversity. To have such an institution in our country is of particular interest since Mexico contains as much as 10% of the world's organismal diversity.

Two new scientific societies focused on genomics have been established: the Mexican Society of Genomic Sciences (http://smcg.ccg.unam.mx/) and the Mexican Society of Genomic Medicine (http://www.somegen.org.mx/). These societies together encompass most of the scientists working on basic or applied genomics in our country.

It should be mentioned that basic research in Mexico is funded mostly by public resources. In particular, UNAM provides a budget that covers the salaries of researchers and technicians, the administration of research, and, most important, a basic budget platform to perform research. In addition, research in genomics and in all other areas depends strongly on grants by CONACYT, the major governmental agency funding science. Public funding also comes through resources from universities, the health sector, and other public institutions. The Mexican Foundation for Health as well as Fundación UNAM provide means to contact private sources (i.e., Fundación Río Arronte and Fundación Ricardo Zevada), which are of great help but much more limited. Philanthropy and fundraising are not established traditions in our country. Funding also comes from foreign agencies such as the US National Institutes of Health, the Howard Hughes Medical Institute, and the European Union. Resources from applied research or patents are almost nonexistent.

Mexican scientists have been able to launch interesting projects, some of them focused on native organisms; to develop bioinformatic resources; to get state-of-the-art equipment and acquire the corresponding methodology; and to establish key institutions. In this way, Mexico has initiated its contribution to genomic sciences. However, this is not at all sufficient. There is a long way to go in terms of enhancing interactions across institutions, in terms of moving toward more integrative genomics, maybe including modeling and systems biology, and toward correlating basic and applied aspects of human genomics, among other areas. In this sense, the most critical limiting step for future development is fostering outstanding human resources.

## Undergraduate Program in Genomics

During 2001, the authors had the privilege to serve, together with other scientists, on a committee appointed by the rector of UNAM, Juan Ramón de la Fuente, with the mission to propose a project to generate the needed human resources for the development of genomic sciences in Mexico. In our view, this project has been the most important action taken in Mexico toward developing genomic sciences. We will dedicate the rest of this article to analyzing this project. (For author information, see Box 1.)

**Box 1. Authors' Biographies**

**Dr. Rafael Palacios de la Lama**, researcher at the Universidad Nacional Autónoma de México, Morelos, is a foreign member of the National Academy of Sciences (USA) and one of the world's experts on the genetics of *Rhizobium* and genome dynamics. He obtained his PhD from the National University of Mexico in 1970 and went on to a postdoctoral position at Stanford University. Since returning to Mexico, he has pioneered the introduction of modern molecular biology there. Palacios has spent several years investigating the genetics of *Rhizobium* and has shown that the genome of these bacteria contains segments that can be rearranged in the laboratory to produce more effective variants of the bacteria. In 2003, he was awarded the annual Third World Academy of Sciences (TWAS) prize, which ranks among the highest scientific accolades given to scientists in developing countries. He was invited to join the NAS in 2006. Palacios' research also focuses on education and the development of the relatively new field of genomic sciences in Mexico. “Lo que me gustaría comunicar a los jóvenes es que se puede hacer buena ciencia en México.”

**Dr. Julio Collado-Vides,** a leader in understanding regulation of the complete E. coli genome, is currently a professor at and the Director of the Center for Genomic Sciences (CCG) of the Universidad Nacional Autónoma de México (UNAM). He is a pioneer in the development of genomics and bioinformatics in the country and renowned as an international leader on the topics. Upon graduation from UNAM, he was at the Department of Biology at the Massachusetts Institute of Technology for three years with an International Fogarty postdoctoral Fellowship. He was President/Founder of the Mexican Society of Genomics (2000) and is currently a regular member of the Scientific Research Academy as well as in charge of the Bioinformatics National Node EMBNET. In an attempt to take genomics teaching to the high school level, he has promoted courses addressed to the professors of UNAM's National High School by a group of investigators from the CCG.

The initial discussions of the committee, formed by scientists from CCG and IBT, were centered on the following issues. Where should students be trained, in Mexico or abroad? Should we train each student in the combined disciplines required by genomic sciences, or should we train students in specific disciplines and expect to form interdisciplinary research teams? At what stage should training begin, at the undergraduate level or the graduate level?

After intensive discussions, we arrived at the following conclusion: we should establish an Undergraduate Program in Genomic Sciences (LCG; http://www.lcg.unam.mx/) at UNAM devoted to training each student in the different disciplines involved in genomic sciences: mathematics, statistics, computer sciences, and biology. This training would continue with a Ph.D. program that could be undertaken in Mexico or abroad, and would culminate with a postdoctoral training in a laboratory at the international level in specific aspects of genomic sciences. The LCG was approved by the University Council in 2003. It is located on the Morelos campus of UNAM in Cuernavaca, and it functions as a joint responsibility of CCG and IBT, with the collaboration of other research and teaching institutions at UNAM (School of Medicine, Institute for Biomedical Research, Institute of Cellular Physiology, Institute of Mathematics, and Institute of Physical Sciences).

The program of LCG comprises nine semesters. It is divided in two levels: basic and professional. The basic level (seven semesters) covers the essential elements of genomic sciences. It is shaped by five thematic fields: mathematics, computer sciences and statistics, structural genomics and evolution, functional genomics, and research seminars. The programs of the first four thematic fields start with basic concepts in each area, and then progress to more integrative concepts joining the different fields. The research seminars allow students to discuss recent progress in genomics with leaders in the field, from both Mexico and different laboratories around the world. Videoconferencing enables the participation of experts at remote places to enrich regular courses and seminars. This has definitely been helpful, particularly in the final courses of the program. These experiences add to those of the fifth and sixth semesters, when students have a weekly seminar with invited scientists, leaders in different aspects of genomics at the international level from first-class institutions. Our guests also interact with researchers and present lectures that are transmitted via videoconference to different institutions in Mexico. This program, known as “Frontiers in Genomics” (http://www.lcg.unam.mx/frontiers/), is a joint collaboration of CCG, IBT, LCG, and the Mexican Society for Genomic Sciences, and has received generous support from the Howard Hughes Medical Institute.

Each year, a new cohort of 30–40 students is incorporated. The first cohort, which started in August 2003, will finish its undergraduate training next October. There has been a big demand for this program: the most recent cohort, which started in August 2006, consists of 35 students selected out of 400 applicants. A building for LCG was constructed at CCG, where each cohort has a classroom with individual computer terminals connected to a powerful SUN server. Each classroom is designed and equipped as a videoconference auditorium. In addition, research laboratories, genomics technical units, and libraries are open to the students at both CCG and IBT.

LCG greatly depends on the continual cooperation of institutions and researchers from abroad. In particular, the program needs international cooperation for the following activities: participation of international leaders in videoconferences or as invited lecturers to present seminars and to discuss research with students and faculty members, and admission of students into international laboratories or industries to perform research during two-month summer periods or the semesters corresponding to the professional level (eight and ninth semesters; see above), or at a later stage, as Ph.D. students or postdoctoral fellows. To facilitate this cooperation, UNAM and, in particular, CCG and IBT will promote formal agreements with international institutions and researchers.

This undergraduate program, unique in our country, and to our knowledge one of the few in the world, is being continually evaluated by the international researchers who have interacted with the students through seminars and discussions. Based on their opinions, we can state that LCG is a very successful program and that the students are highly motivated and talented and are obtaining excellent training. These students, together with those from other training programs at the graduate level at UNAM and CINVESTAV, make us feel highly optimistic with regard to the future of genomic sciences in Mexico. 

## Abbreviations

CCG: Center for Genomic Sciences
CIFN: Nitrogen Fixation Research Center
CINVESTAV: Center for Research and Advanced Studies
CONACYT: National Council of Science and Technology
IBT: Institute of Biotechnology
LCG: Undergraduate Program in Genomic Sciences
UNAM: National Autonomous University of Mexico
